# A simplified isolation technique for atherosclerotic aortic arch aneurysms surgery

**DOI:** 10.1093/jscr/rjab082

**Published:** 2021-04-19

**Authors:** Yuta Kikuchi, Yoichi Kikuchi, Hiroyuki Kamiya

**Affiliations:** 1 Department of Cardiovascular Surgery, Asahikawa Medical University, Asahikawa, Hokkaido, Japan; 2 Department of Cardiovascular Surgery, National Hospital Organization Obihiro Hospital, Obihiro, Hokkaido, Japan

## Abstract

The isolation technique is a useful adjunct that prevents atherosclerotic embolism in the brain when the aneurysm is filled with a massive hematoma or ‘shaggy aorta’. But the technique is not widespread because of the difficulty in performing the cannulation. We modified this technique by simplifying the cannulation procedure using a puncture method with aortic root cannulas.

## INTRODUCTION

Antegrade selective cerebral perfusion has become the mainstream adjunct for brain protection in aortic arch surgery. Various methods have been reported [1–3], such as perfusion of arch vessels with balloon catheters and direct cannulation to arch vessels with a small aortic perfusion cannula. Among these techniques, the isolation technique, introduced in 2001, is a useful adjunct that prevents atherosclerotic embolism in the brain when the aneurysm is filled with a massive hematoma or ‘shaggy aorta’ [4]. However, this technique is not widespread because of the increased probability of bleeding during cannulation and the difficulty in performing the cannulation. We modified this technique by simplifying the cannulation procedure using a puncture method with aortic root cannulas.

## TECHNIQUE

**Figure 1:**
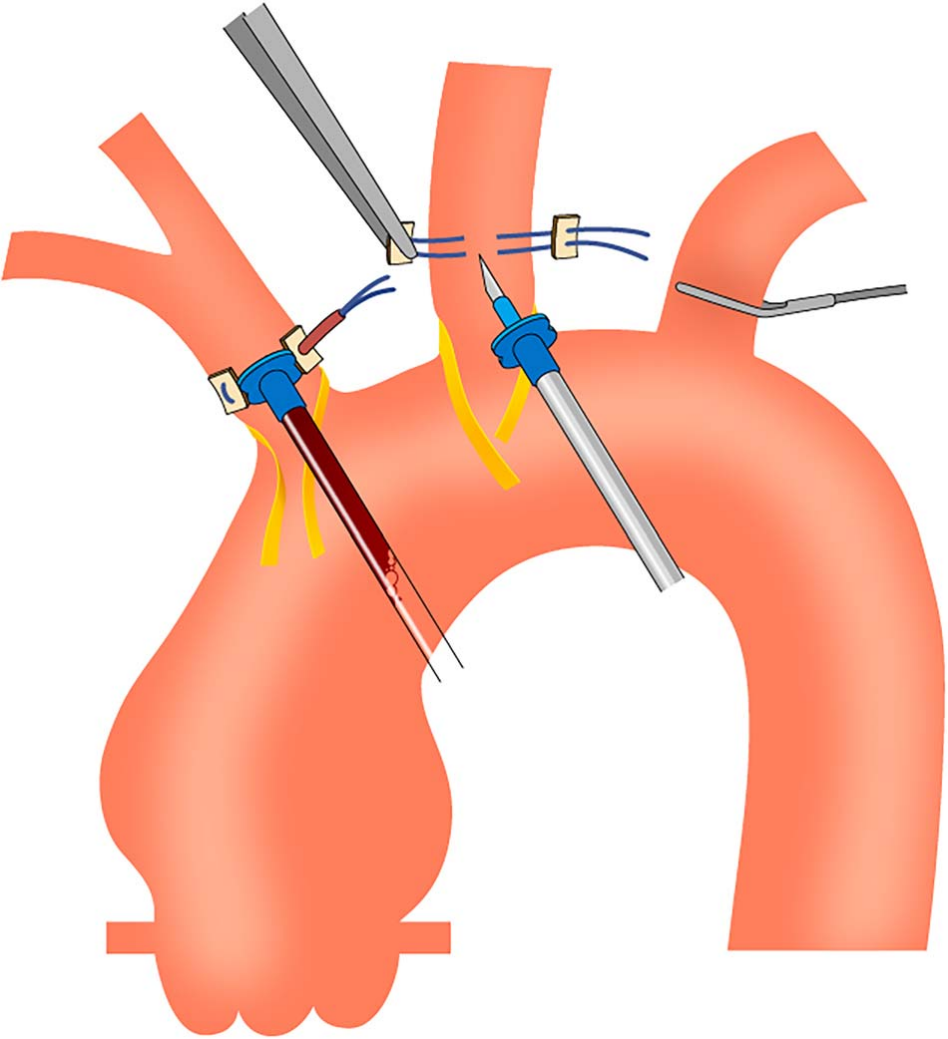
Schema of the simplified isolation technique using aortic root cannulas.

**Figure 2:**
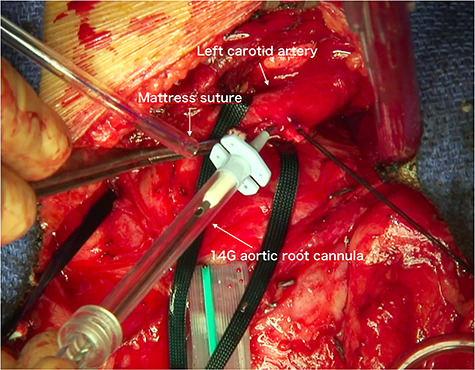
Photograph obtained during surgery. The 14G aortic root cannula is seen puncturing the left common carotid artery.

During the anesthesiologic preparation, small pressure catheters were inserted into the right radial artery, left radial artery and left femoral artery. Bilateral cerebral oxygen saturation was monitored by near-infrared spectroscopy. After median sternotomy, the arch vessels were encircled with tape. The ascending aorta, arch vessels and aortic arch were examined using an epiaortic echogram to check for intraluminal hematoma and sclerotic changes in the aortic wall. We routinely adopt two-vessel perfusion for selective cerebral perfusion of the brachiocephalic artery and left carotid artery; thus, 6–0 polypropylene mattress sutures with small pledgets were used for these vessels. Cardiopulmonary bypass was established with aortic cannulation of the ascending aorta and venous cannulation of the right atrium. Immediately after the cardiopulmonary bypass was initiated, aortic root cannulas were inserted into these vessels (Figs 1 and 2). Subsequently, cerebral perfusion was initiated. We used 12G and 14G aortic root cannulas for the brachiocephalic and left carotid arteries, respectively. We continued to keep the body cool at 25°C, and during the cooling period, the ascending aorta was cross-clamped and transected, and the proximal aorta was reinforced with a felt strip. At this point, the arch vessels were not clamped because the safety of normothermic brain perfusion is uncertain. Moreover, the atherosclerotic embolism might block the bloodstream from the cerebral perfusion line to the arch vessels. At rectal temperatures < 30°C, the arch vessels were clamped, and brain perfusion was accomplished. Blood flow was initially set at 400 ml/min in the brachiocephalic artery and 200 ml/min in the left carotid artery. The flow in the brachiocephalic artery was adjusted to maintain the arterial pressure at the right radial artery at ~50 mmHg, and the right-sided cerebral oxygen saturation was ~60%. The flow in the left carotid artery was adjusted to maintain the pressure in the circuit at ~150 mmHg, and the left-sided cerebral oxygen saturation was ~60%. At a rectal temperature of 25°C, systemic circulation was discontinued, and the aortic arch was opened. We routinely use a four-branched woven Dacron graft to reconstruct the aortic arch. First, distal anastomosis was performed, followed by reconstruction of the left subclavian artery. The systemic circulation was resumed from the side branch of the graft, and the body was rewarmed. We then performed a proximal anastomosis and released the aortic cross-clamp. Next, we reconstructed the left carotid artery and brachiocephalic artery. After evacuating the air, the aortic root cannulas were removed by simply tying down the 6–0 polypropylene sutures.

We also used this method for hemiarch replacement, which requires cerebral perfusion. The commonly used balloon catheter is cumbersome during distal anastomosis, whereas our technique makes it possible to perform distal anastomosis at any site.

In our institution, we have thus far employed this technique for six aortic arch replacement and three hemiarch replacement cases. Mean selective cerebral perfusion time, hypothermic circulatory arrest time, aortic cross-clamping time and cardiopulmonary bypass time were 81.3 ± 2.7 min, 63.2 ± 4.1 min, 75 ± 2.4 min and 123.8 ± 5.4 min, respectively. There were no mortalities or neurological complications.

## DISCUSSION

Many superior studies favoring selective cerebral perfusion with moderate or deep hypothermia as an adjunct for protection of the brain during an aortic arch aneurysm repair have been reported in this decade. In particular, the isolation technique, an alternative method for preventing cerebral infarction due to thromboembolism from the aortic arch, is advantageous when there is a severe atherosclerotic change in the aortic arch and arch vessels. However, this technique involves possible bleeding complications and a temporary decrease in cerebral blood flow. In addition, whether normothermic perfusion to the brain isolated from systemic circulation is safe is still unclear. Therefore, this technique is not widely used. In our modified and simplified isolation technique, we insert the perfusion cannula via a puncture, thus minimizing bleeding complications and preventing the reduction of blood flow to the brain. We used 12G and 14G aortic root cannulas for the brain perfusion. A problem associated with this technique is the potential risk of posterior wall injury if the arch vessels are too small. Due to the relatively small number of cases studied, further research is essential to evaluate the superiority of this technique over other selective cerebral perfusion techniques.
